# Kounis Syndrome in Cardiac Surgery: Pathophysiology, Antimicrobial Triggers, and Perioperative Recognition and Management

**DOI:** 10.3390/medsci14020207

**Published:** 2026-04-23

**Authors:** Vasileios Leivaditis, Christodoulos Chatzigrigoriadis, Efstratios Koletsis, Virginia Mplani, Periklis Dousdampanis, Francesk Mulita, Nicholas G. Kounis, Stelios F. Assimakopoulos

**Affiliations:** 1 Department of Cardiothoracic and Vascular Surgery, Westpfalz Klinikum, 67655 Kaiserslautern, Germany; 2 Department of Internal Medicine, Division of Infectious Diseases, General University Hospital of Patras, 26504 Patras, Greece; up1084142@ac.upatras.gr (C.C.); sassim@upatras.gr (S.F.A.); 3 Department of Cardiothoracic Surgery, General University Hospital of Patras, 26504 Patras, Greece; ekoletsis@hotmail.com; 4 Intensive Care Unit, General University Hospital of Patras, 26504 Patras, Greece; virginiamplani@yahoo.gr; 5 Department of Nephrology, Saint Andrews State General Hospital, 26221 Patras, Greece; dousdampanis@yahoo.gr; 6 Department of General Surgery, General Hospital of Eastern Achaia–Unit of Aigio, 25100 Aigio, Greece; med5507@ac.upatras.gr; 7 Department of Internal Medicine, Division of Cardiology, General University Hospital of Patras, 26504 Patras, Greece; ngkounis@otenet.gr

**Keywords:** Kounis syndrome, allergic acute coronary syndrome, allergic angina, perioperative anaphylaxis, coronary vasoconstriction, cardiac surgery, cardiopulmonary bypass, extracorporeal membrane oxygenation, coronary artery bypass graft, valve replacement, perioperative anaphylaxis, chlorhexidine, protamine, cefazolin, cefuroxime, beta-lactam, vancomycin, teicoplanin, transesophageal echocardiography

## Abstract

Background: Kounis syndrome is an allergic acute coronary syndrome precipitated by coronary vasospasm, plaque destabilization, stent thrombosis, or bypass occlusion. Cardiac surgery represents a uniquely high-risk setting due to cardiopulmonary bypass–associated inflammation and exposure to multiple pharmaceutical agents. Importantly, Kounis syndrome remains underrecognized in this context, as classical signs of anaphylaxis may be masked under general anesthesia and cardiopulmonary bypass, while ischemic manifestations may be misattributed to other perioperative conditions. Methods: A narrative review of PubMed-indexed literature was conducted to synthesize current evidence on the pathophysiology, perioperative triggers, clinical presentation, diagnostic strategies, and management of Kounis syndrome in cardiac surgery, with emphasis on intraoperative recognition and surgical decision-making. Published cases were retrieved involving perioperative cardiac surgery patients with a definite diagnosis of Kounis syndrome. Additionally, cases presenting with severe perioperative anaphylaxis and life-threatening cardiovascular involvement (grade III with cardiovascular collapse and grade IV with cardiac arrest) were included as possible Kounis syndrome, reflecting real-world diagnostic uncertainty in the intraoperative setting. Results: The literature review identified five cases of definite Kounis syndrome and ten cases of possible Kounis syndrome, including three cases with cardiovascular collapse and seven cases with cardiac arrest. Recurrent episodes were reported in several patients, particularly due to re-exposure to the triggering agent. In the context of cardiac surgery, Kounis syndrome is most frequently triggered by chlorhexidine, protamine, antibiotic prophylaxis, and anesthetic agents. The clinical presentation is often subtle during cardiopulmonary bypass. Vasoplegia, pulmonary hypertension, ventricular dysfunction, new regional wall-motion abnormalities, and hyperdynamic ventricles on transesophageal echocardiography commonly precede overt electrocardiographic changes. Diagnosis is primarily clinical and relies on intraoperative ultrasound, hemodynamic monitoring, serum tryptase, serum troponin, and, when indicated, coronary angiography. A dual-pathway approach addressing both anaphylaxis and myocardial ischemia is essential; however, one component may predominate, particularly in perioperative patients with limited clinical information, potentially leading to misdiagnosis. A multidisciplinary approach is therefore required for rapid diagnosis and individualized management. In refractory cases, cardiopulmonary bypass or ventricular assist devices may provide lifesaving support. Conclusions: Kounis syndrome remains underrecognized in cardiac surgery but carries significant morbidity. Increased clinical awareness, multidisciplinary collaboration, structured diagnostic approaches, and preventive strategies are essential to improve outcomes and reduce the risk of recurrence during future procedures.

## 1. Introduction

Kounis syndrome, also known as allergic angina or allergic myocardial infarction (MI), was first conceptualized by Kounis and Zavras in 1991 and describes the occurrence of acute coronary events triggered by hypersensitivity reactions [1]. Immune-mediated cardiac and vascular inflammation most commonly occurs in the context of anaphylaxis but has also been reported in association with other systemic disorders, including serum sickness, serum sickness-like reactions, systemic lupus erythematosus, and acquired immunodeficiency syndrome [2,3,4,5]. Although primarily affecting the coronary circulation, Kounis syndrome may rarely involve other vascular territories and organs [6,7,8,9,10,11,12,13]. The syndrome is characterized by mast cell activation and the subsequent release of inflammatory and vasoactive mediators, leading to coronary vasospasm (type I), atherosclerotic plaque disruption (type II), or coronary stent or bypass occlusion (type III/IV) [14,15,16]. Over the past decades, advances in basic and clinical research have established that immunologic and cardiovascular processes are closely interconnected through mast cell activation and should not be considered independent clinical entities [17,18,19,20,21,22,23].

Mast cells play a central role in the pathogenesis of Kounis syndrome, in a manner analogous to anaphylaxis. The classical mechanism is immunoglobulin E (IgE)-mediated, in which allergens interact with specific IgE molecules bound to the high-affinity Fc epsilon receptor I (FcεRI) on the surface of mast cells [24,25,26]. However, non-IgE-mediated pathways have also been described, including complement activation with the formation of anaphylatoxins (C3a, C5a), immunoglobulin G (IgG) binding to Fc gamma receptor I (FcγRI), and direct mast cell activation via the Mas-related G protein-coupled receptor X2 (MRGPRX2) [24,25,26,27]. Following activation, mast cells release a wide range of inflammatory mediators, such as histamine, chymase, arachidonic acid metabolites, tryptase, and platelet-activating factor [15,25,26,28]. These mediators induce endothelial dysfunction, smooth muscle contraction, and platelet activation, resulting in coronary vasoconstriction, thrombosis, and potential plaque rupture [15,25,26,28]. Consequently, myocardial hypoperfusion may lead to arrhythmias, impaired contractility, and hemodynamic instability due to concomitant systemic vasodilation [15,25,29]. Importantly, Kounis syndrome may occur in patients with either normal coronary arteries or pre-existing coronary artery disease, reflecting its broad clinical spectrum [10,24,25,30].

Although anaphylaxis and Kounis syndrome occur in various clinical settings, they may also arise in the perioperative environment. Perioperative stress is known to promote mast cell activation, potentially increasing the risk of hypersensitivity reactions and myocardial ischemia [31]. Perioperative anaphylaxis is a well-recognized but relatively rare condition, most commonly triggered by pharmacologic or material exposures, including antibiotics, neuromuscular blocking agents (NMBAs), sugammadex, chlorhexidine, dyes, and latex [32,33,34,35,36,37,38,39]. Numerous reports have also described perioperative Kounis syndrome associated with similar agents, with particular relevance in cardiac surgery [10,30,40,41,42,43,44,45,46,47,48,49,50,51,52]. Cardiopulmonary bypass (CPB) induces a profound systemic inflammatory response through blood–surface interactions, complement activation, cytokine release, endothelial injury, and activation of the coagulation cascade. However, factors such as hypothermia, hemodilution, and heparin may partially modulate this response [41,53,54]. Within this highly sensitized perioperative environment, allergic reactions may be amplified and can directly translate into myocardial ischemia.

Among pharmacologic triggers, perioperative antibiotic prophylaxis—particularly with agents such as cefazolin or vancomycin—represents one of the most common causes of anaphylaxis and has also been implicated in Kounis syndrome during cardiac surgery [36,40,55,56,57,58,59,60]. This association highlights the intersection between infectious diseases, allergy, anesthesiology, and cardiovascular medicine, underscoring the importance of accurate allergy history, appropriate antibiotic selection, and vigilant intraoperative monitoring [61]. Additional triggers, including protamine for heparin reversal and chlorhexidine used for infection prevention, further contribute to the risk during cardiac surgical procedures [47,58,62,63,64,65,66,67].

Despite increasing recognition through case reports, mechanistic studies, and perioperative audits linking allergic pathways to coronary injury, Kounis syndrome remains underdiagnosed in the intraoperative setting, and its true incidence in cardiac surgery is likely underestimated [40]. Classical manifestations of anaphylaxis, such as rash and bronchoconstriction, may be masked by general anesthesia and CPB; similarly, ischemic signs, such as electrocardiographic (ECG) abnormalities and wall motion abnormalities (WMAs), may be misattributed to vasoplegia or reperfusion injury rather than an underlying allergic mechanism [28,40,41,52,56]. These diagnostic limitations contribute to uncertainty in perioperative recognition and highlight the lack of structured approaches for timely diagnosis and management in this high-risk setting. Thus, a thorough postoperative review of the patient’s history and perioperative audit, along with laboratory tests, electrocardiogram, echocardiogram, and coronary angiography, can help identify missed cases and reduce suboptimal management and perioperative morbidity [24,28,40,41,56,65,66,67,68].

This review presents current evidence on Kounis syndrome with a specific focus on cardiac surgery, synthesizing PubMed-indexed literature to elucidate pathophysiology, common perioperative triggers, diagnostic challenges, and management considerations relevant to the cardiac surgical team. The aim of this review is to synthesize current evidence on Kounis syndrome in cardiac surgery, with a particular focus on perioperative pathophysiology, diagnostic challenges, and clinically applicable management strategies to support intraoperative recognition and decision-making. The rarity of perioperative Kounis syndrome limits the available literature, which explains the lack of observational studies and clinical trials; hence, multiple case reports and case series were used to extract conclusions.

## 2. Materials and Methods

### 2.1. Study Design

This study was designed as a narrative literature review of published perioperative cases and relevant literature addressing the clinical relevance of Kounis syndrome in the setting of cardiac surgery. The review focuses on perioperative pathophysiology, common triggers, diagnostic challenges, and management considerations specific to cardiothoracic surgical practice. As the study was based exclusively on previously published data, ethics committee approval and informed consent were not required.

### 2.2. Literature Search Strategy

A comprehensive literature search was conducted using PubMed/Medline, screening publications up to January 2026. The literature search was performed using a combination of free-text terms and Medical Subject Headings (MeSH), where applicable. Screening was conducted based on titles and abstracts, followed by full-text assessment of potentially relevant articles. No strict lower date limit was applied due to the rarity of the condition, and all available literature up to January 2026 was considered eligible.

The search strategy employed a combination of: “(Kounis syndrome OR allergic myocardial infarction OR allergic angina OR perioperative anaphylaxis)” AND (cardiac surgery OR cardiothoracic surgery OR cardiopulmonary bypass OR transesophageal echocardiography OR cefazolin OR valve replacement OR coronary artery bypass graft OR protamine OR vancomycin OR teicoplanin OR perioperative prophylaxis OR antibiotic prophylaxis OR tryptase OR ciprofloxacin OR ampicillin-sulbactam OR piperacillin-tazobactam OR central venous catheter OR quinolone OR b-lactam OR chlorhexidine OR infective endocarditis OR aortic dissection OR aortic aneurysm OR coronary artery aneurysm OR aortic dilation OR aneurysm repair OR heart transplant OR cardiac transplant OR left ventricular assistant device)”.

The search was intentionally broad to capture mechanistic, clinical, and perioperative perspectives relevant to cardiac surgery. In addition, reference lists of key publications were manually screened to identify further pertinent articles not retrieved in the initial search.

### 2.3. Eligibility Criteria

Publications were considered eligible if they met the following criteria:

1. Description of patients with a combination of anaphylaxis and myocardial ischemia, which represents a definite diagnosis of perioperative Kounis syndrome.
2. Description of patients with perioperative anaphylaxis and life-threatening cardiovascular involvement, such as cardiovascular collapse (grade III) and cardiac arrest (grade IV). These cases are considered possible cases of perioperative Kounis syndrome despite the lack of clear evidence of coronary hypoperfusion.
3. Relevant to cardiac surgery, including coronary artery bypass graft (CABG), valve replacement, and large vessel pathology.
4. Various types of articles, including case reports, case series, and observational studies.
5. Available full-text.
6. English language.

The inclusion of cases classified as “possible” Kounis syndrome was intended to increase sensitivity and capture clinically relevant perioperative scenarios in which definitive diagnostic confirmation is often limited by intraoperative conditions. These cases were analyzed separately and interpreted with caution to avoid overestimation of definite Kounis syndrome.

### 2.4. Data Selection and Synthesis

Given the narrative nature of this review, study selection and data interpretation were performed qualitatively, without formal risk-of-bias assessment or meta-analytic techniques.

Articles were reviewed for relevance to cardiac surgery, perioperative timing, identified triggers, clinical presentation, diagnostic modalities, and management strategies. Given the heterogeneity of study designs and the predominance of case-based literature, a qualitative synthesis was performed rather than a quantitative meta-analysis. The following variables were extracted and presented: epidemiological characteristics of the patients (age, sex, type of surgery), trigger, onset, clinical presentation, investigation, and treatment. Emphasis was given to patterns and concepts directly applicable to clinical decision-making in cardiac surgical practice.

Relevance was assessed based on the presence of perioperative timing, compatibility of clinical presentation with hypersensitivity and/or myocardial ischemia, identification of a potential trigger, and the availability of sufficient clinical detail to support interpretation.

### 2.5. Methodological Limitations

This review has inherent methodological limitations. The search strategy was intentionally broad to capture the limited and heterogeneous literature available on perioperative Kounis syndrome in cardiac surgery; however, it was not conducted as a systematic review and may therefore be subject to selection bias. The absence of a predefined lower date limit and reliance on PubMed-indexed literature may have resulted in the omission of relevant studies from other databases or unpublished sources. Furthermore, the inclusion of cases classified as “possible” Kounis syndrome—defined by severe perioperative anaphylaxis with life-threatening cardiovascular involvement—was intended to reflect real-world clinical uncertainty but may have reduced diagnostic specificity and potentially inflated the number of reported cases. To mitigate this limitation, these cases were clearly distinguished from definite cases and interpreted cautiously. Finally, heterogeneity in case definitions, diagnostic approaches, and reporting across the included studies limits comparability and precludes quantitative synthesis.

## 3. Results

For clarity, cases are presented according to diagnostic certainty, distinguishing between definite and possible Kounis syndrome. This distinction was maintained throughout the analysis to ensure clarity in the interpretation of findings. A total of 13 publications were included, presenting 15 patients with the diagnosis of perioperative Kounis syndrome or anaphylaxis with life-threatening cardiovascular involvement. These were 12 case reports and one case series with 3 patients. A definite diagnosis of perioperative Kounis syndrome was established in 5/15 patients, while the remaining 10/15 patients were classified as possible Kounis syndrome, presenting with anaphylaxis and severe cardiovascular involvement (cardiovascular collapse in three cases and cardiac arrest in seven cases). Thus, these additional cases can be classified as possible Kounis syndrome.

Most procedures were elective (11/15). The most common types of surgery were coronary artery bypass grafting (CABG) (6/15) and mitral valve replacement (MVR) (5/15). Most patients were middle-aged or elderly (50–79 years), with only a few younger cases reported.

The identified triggers of perioperative Kounis syndrome in cardiac surgery were chlorhexidine (6/15), protamine (2/15), amiodarone (2/15), colloids (2/15), rocuronium (1/15), and cefuroxime (1/15), listed in descending order of frequency.

All cases of chlorhexidine-induced Kounis syndrome developed within minutes of the placement of a chlorhexidine-coated central venous catheter (CVC) during surgical preparation. In one case, simultaneous skin application of chlorhexidine preceded the reaction. Notably, most patients (5/6) experienced recurrent episodes due to delayed identification of the trigger and repeated exposure. A history of prior skin hypersensitivity to chlorhexidine was reported in two cases.

Both protamine-induced cases occurred shortly after cessation of cardiopulmonary bypass (CPB) and heparin reversal, typically following completion of the procedure. One of these patients experienced recurrent postoperative episodes related to repeated protamine administration.

Amiodarone-induced perioperative Kounis syndrome was recognized early during the management of intraoperative arrhythmias in both reported cases, and no recurrences were observed.

Kounis syndrome associated with colloid administration developed rapidly during infusion. In one case, a reaction occurred at the end of the operation during fibrinogen concentrate administration and recurred postoperatively during blood transfusion; albumin was identified as the causative agent. Another case was triggered by gelatin colloid (Gelaspan), resulting in fatal anaphylaxis despite intensive care management.

A case of perioperative Kounis syndrome related to the neuromuscular blocking agent rocuronium occurred during induction of general anesthesia and was promptly diagnosed without recurrence. In another case, anesthetic agents (rocuronium or isoflurane) were suspected to be responsible following retrospective evaluation, with a protracted postoperative course.

Finally, one case of perioperative Kounis syndrome secondary to antibiotic prophylaxis (cefuroxime) was identified shortly after administration, without recurrence. Among pharmacologic triggers, antimicrobial agents—particularly beta-lactam antibiotics such as cefuroxime—represent clinically important causes of perioperative Kounis syndrome, despite being underrepresented in the limited number of reported cases.

The clinical presentation and diagnostic findings varied considerably across cases and frequently involved multiple organ systems. Cardiovascular involvement was present in all cases and manifested as hemodynamic instability, including bradycardia or tachycardia, hypotension, and/or myocardial ischemia. Cardiac arrest—either pulseless electrical activity or ventricular fibrillation—occurred in 2/5 cases of definite Kounis syndrome and 7/10 cases of possible Kounis syndrome.

Electrocardiographic abnormalities were common and included ST-segment elevation, ST-segment depression, and atrial fibrillation. Elevation of cardiac biomarkers (troponin, creatine kinase, and creatine kinase–myocardial band) was observed in 3/15 cases. Echocardiographic abnormalities were reported in five cases and included biventricular dysfunction, hyperdynamic ventricles, regional wall motion abnormalities, and apical ballooning consistent with Takotsubo syndrome.

Coronary angiography—performed invasively in three patients and via computed tomography in one—demonstrated generalized vasospasm in two cases and normal findings in the remaining two cases.

Pulmonary involvement was either subclinical or clinically evident. Subclinical manifestations were suspected intraoperatively based on a sudden decrease in end-tidal carbon dioxide (EtCO_2_) and increased airway pressures in three cases. In contrast, four cases showed overt respiratory involvement, including bronchospasm or dyspnea.

Mucocutaneous manifestations were observed in several cases, with rash reported in eight patients and angioedema in three; two patients exhibited both. Notably, these findings were consistently present in only two patients, while six patients with recurrent episodes exhibited mucocutaneous signs in only one episode.

Assessment of allergy biomarkers revealed elevated tryptase levels in ten cases. Two of these cases also showed increased immunoglobulin E (IgE), and one case demonstrated eosinophilia. Normal tryptase levels were reported in two cases; however, both measurements were obtained with delay. Further allergological evaluation identified the causative agent in 10/15 cases, primarily through skin testing (8 cases) and, less frequently, specific IgE measurement (2 cases).

Treatment strategies were heterogeneous and largely guided by clinical presentation and timing, but were primarily directed toward the management of anaphylaxis and cardiovascular instability. Adrenaline was administered in the majority of cases (14/15). Additional therapies included inhaled β2-agonists (3 cases), antihistamines (8 cases), and corticosteroids (8 cases). Various vasopressors—including noradrenaline, phenylephrine, vasopressin, desmopressin, calcium chloride, chlorpheniramine, metaraminol, methylene blue, and ephedrine—were used in most cases (11/15) to maintain hemodynamic stability. Inotropic support with agents such as dobutamine and isoprenaline was required in five cases. Nitrates were used in one case to reverse coronary vasoconstriction.

Advanced circulatory support was required in several cases, including cardiopulmonary bypass (CPB) (3/15), extracorporeal membrane oxygenation (ECMO) (3/15), and biventricular assist device (BiVAD) support (1/15). CPB was used for rapid stabilization to allow continuation of surgery in one case and during completion of the procedure in two additional cases. ECMO was initiated at the start of surgery in one patient, continued postoperatively in a case of Takotsubo syndrome, and later reintroduced in another patient as a bridge to BiVAD support.

Overall, outcomes were generally favorable. Most patients survived due to early recognition of circulatory shock, respiratory failure, or cardiac arrest in the operating room or intensive care unit, allowing prompt initiation of supportive measures. One fatal case was reported in a critically ill patient who developed refractory anaphylaxis unresponsive to vasopressor therapy.

Surgical procedures were successfully completed in several cases despite intraoperative instability, including one case following cefuroxime administration and three cases after insertion of chlorhexidine-coated central venous catheters. In contrast, surgery was initially aborted in four cases due to life-threatening allergic reactions—three following chlorhexidine exposure and one after induction with rocuronium. These patients subsequently underwent repeat surgery, with only one unsuccessful attempt. Repeat surgical intervention was also required in three cases to exclude complications related to the surgical field and in one case for BiVAD implantation.

For clarity and ease of interpretation, a structured summary of the reported cases—including classification (definite vs. possible Kounis syndrome), triggers, clinical characteristics, management, and outcomes—is presented in Table 1.

## 4. Discussion

### 4.1. Pathophysiology of Perioperative Kounis Syndrome

The heterogeneity of intraoperative presentations of Kounis syndrome reflects the underlying coronary substrate and the pattern of immune activation. Type I Kounis syndrome is defined by coronary vasospasm in angiographically normal vessels, whereas type II is associated with vasoconstriction and/or allergic plaque rupture in the presence of atherosclerosis [17,69]. Type III Kounis syndrome is characterized by stent thrombosis, supported by histopathologic evidence of inflammatory cell infiltration within the thrombus and adjacent coronary vessel, highlighting the direct link between allergic inflammation and coronary thrombosis [17,69,70,71]. This type is further subdivided into stent thrombosis (type IIIa) and stent restenosis (type IIIb) [6,10]. Type IV Kounis syndrome, the most recently described variant, affects patients with prior coronary artery bypass grafting (CABG) and is defined by graft occlusion [14,16,72]. In the cardiac surgical population—often characterized by underlying coronary artery disease, prior percutaneous coronary intervention (PCI), and repeated antigen exposure—this classification is particularly relevant and adds complexity to intraoperative decision-making.

Perioperative Kounis syndrome represents the convergence of allergic inflammation, endothelial dysfunction, and platelet–vascular interactions within the highly proinflammatory environment of cardiac surgery, especially during cardiopulmonary bypass (CPB). Both immunoglobulin E (IgE)-mediated hypersensitivity reactions and non–IgE-mediated mechanisms—such as direct mast cell activation and complement-mediated pathways—can trigger mast cell degranulation [24,25,73]. The subsequent release of inflammatory mediators, including histamine, tryptase, chymase, leukotrienes, prostaglandins, cytokines, tumor necrosis factor-α, and platelet-activating factor, leads to coronary vasoconstriction, microvascular dysfunction, increased vascular permeability, and the development of a prothrombotic endothelial phenotype [10,15,25,74]. Notably, diffuse epicardial and microvascular spasm may occur even in angiographically normal coronary arteries [17,68,74,75]. In parallel, platelet activation is enhanced through interactions between inflammatory mediators and platelet Fc receptors, promoting both thrombosis and further mediator release [24]. This mechanism provides a direct link between allergic inflammation and acute coronary thrombosis, particularly in type III Kounis syndrome, and explains the presence of thrombi enriched with eosinophils and mast cells within coronary stents [10,70].

Cardiopulmonary bypass induces a complex systemic inflammatory response characterized by complement activation, leukocyte priming, cytokine release, and endothelial dysfunction. This inflammatory milieu amplifies the vascular and myocardial effects of mast cell-derived mediators, potentially lowering the threshold for the development of Kounis syndrome during cardiac surgery. Blood–surface interactions within the extracorporeal circuit lack the regulatory properties of the endothelium, resulting in activation of the complement cascade and the generation of anaphylatoxins such as C3a and C5a, which directly stimulate mast cells and enhance vascular reactivity [76]. In addition, activation of the intrinsic coagulation pathway leads to increased bradykinin production via factor XIIa–mediated conversion of prekallikrein to kallikrein, further contributing to complement activation and vascular instability [53,76]. Bradykinin also modulates vascular tone and leukocyte function, reinforcing the inflammatory response. Although further research is required, factors such as heparin, hypothermia, and hemodilution may partially attenuate complement activation while simultaneously impairing platelet function [53,54,76].

**Figure 1:**
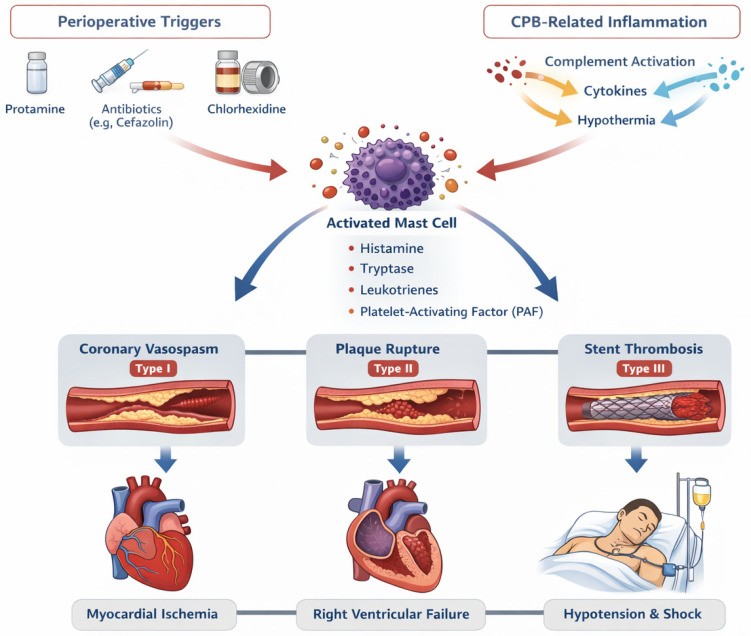
Pathophysiological mechanisms of Kounis syndrome in cardiac surgery.

Collectively, these mechanisms explain why Kounis syndrome in cardiac surgery may present abruptly, progress rapidly, and remain difficult to recognize in its early stages. The interplay between allergic pathways and surgical physiology not only precipitates myocardial ischemia but also complicates intraoperative decision-making. These considerations underscore the need for heightened clinical awareness and a mechanistic understanding of Kounis syndrome among cardiac surgeons and anesthesiologists. The integrated pathophysiology of Kounis syndrome in the cardiac surgical setting is illustrated in Figure 1.

### 4.2. Epidemiology of Perioperative Kounis Syndrome

Perioperative anaphylaxis occurs at a measurable, albeit relatively low, frequency in cardiac surgery, and a clinically relevant subset of these reactions fulfills diagnostic criteria for Kounis syndrome [38,61]. A definite diagnosis requires the coexistence of hypersensitivity features with evidence of myocardial ischemia and/or dysfunction [29,77,78,79]. However, Kounis syndrome may also be considered in patients with anaphylaxis complicated by severe hemodynamic instability (grade III) or cardiac arrest (grade IV) [10,52,61,79]. Despite this framework, the true incidence of perioperative anaphylaxis and Kounis syndrome is likely underestimated [10,52,61,80,81].

Several perioperative factors contribute to underrecognition. Physiological alterations associated with cardiopulmonary bypass (CPB)—including hemodilution, hypothermia, non-pulsatile flow, and deep anesthesia—can obscure the classical cutaneous, respiratory, and hemodynamic signs of allergic reactions [42,44,46,47]. At the same time, myocardial ischemic manifestations are often attributed to alternative perioperative mechanisms, such as supply–demand mismatch, coronary manipulation, air embolism, or reperfusion injury, rather than to an allergic coronary process [42,44,47]. Identification of the causative trigger is often even more challenging than establishing the diagnosis itself, as patients undergoing cardiac surgery are exposed to multiple agents with allergic potential [45,47,49]. The potential synergistic effects of these exposures may further promote mast cell activation and hypersensitivity reactions [43,49].

In the present analysis, perioperative Kounis syndrome was most commonly triggered by chlorhexidine-coated central venous catheters, followed by protamine for heparin reversal after CPB, amiodarone used for arrhythmia management, colloid transfusion, rocuronium during anesthesia induction, and cefuroxime administered as antibiotic prophylaxis. Evidence from large perioperative audits and multicenter observational studies consistently identifies antibiotics, neuromuscular blocking agents, latex, and chlorhexidine as the most frequent intraoperative sensitizers, although their relative contribution varies across institutions and clinical protocols [39,61,82,83,84,85,86,87,88,89,90,91,92].

Chlorhexidine has emerged as a leading trigger of perioperative hypersensitivity, typically occurring shortly after placement or manipulation of a central venous catheter or, less commonly, following skin preparation before surgical incision [45,46,47,52,61,93,94,95,96,97]. Similarly, heightened vigilance is required during anesthesia induction, particularly with non-depolarizing neuromuscular blocking agents, as well as with succinylcholine and sugammadex [47,48,61,92,97]. Antibiotic prophylaxis represents another critical period of risk at the beginning of surgery, especially with β-lactams such as amoxicillin/clavulanate, cefazolin, and cefuroxime [25,39,40,61,97,98,99,100]. When β-lactams are avoided, alternative agents—including fluoroquinolones, vancomycin, and teicoplanin—may also be implicated [25,40,61,97,99,101,102]. Latex exposure remains an additional potential cause of intraoperative anaphylaxis [38,103,104,105]. Furthermore, protamine should be considered a potential trigger of hypersensitivity reactions following CPB termination [58,67,96,106,107].

These exposure-dependent patterns establish a temporal framework that is highly relevant for intraoperative diagnosis [79,108]. The coexistence of myocardial ischemia, coronary spasm, ventricular dysfunction, unexplained hemodynamic collapse, bronchospasm, impaired gas exchange, pulmonary hypertension, rash, or angioedema in temporal association with these exposures should prompt consideration of perioperative hypersensitivity, including Kounis syndrome, rather than being attributed solely to alternative perioperative causes [79].

The majority of reported patients were middle-aged to elderly males undergoing CABG or valve surgery. Preoperative hypersensitivity to chlorhexidine was documented in two cases. Recurrence of Kounis syndrome occurred in seven patients and was strongly associated with continued or repeated exposure to the causative agent, either during postoperative care or subsequent surgical procedures. From a demographic perspective, reported cases reflect the underlying cardiac surgical population, with a predominance of older male patients and a high burden of cardiovascular risk factors. Repeated exposure to sensitizing agents—such as antiseptics, antibiotics, prior surgical interventions, or implanted coronary devices—combined with a history of allergic disease may contribute to cumulative immune sensitization [45,46,71,109]. This interaction between patient susceptibility, procedural exposure, and immune activation likely plays a central role in both the development and underrecognition of perioperative Kounis syndrome.

From a clinical standpoint, the widespread use of antiseptic agents and antibiotic prophylaxis underscores the importance of close collaboration between infectious diseases specialists, allergists, anesthesiologists, and cardiac surgeons. Accurate identification of true β-lactam hypersensitivity is essential, as mislabeling may lead to the use of alternative agents with reduced efficacy or different allergenic profiles, potentially increasing perioperative risk [5,36,110,111]. Similarly, failure to recognize and remove chlorhexidine-containing devices may result in persistent antigen exposure and recurrent or refractory instability, making source control both a diagnostic and therapeutic priority that should be incorporated into perioperative management protocols [45,46,47,52,112].

Overall, the available epidemiological data suggest that perioperative Kounis syndrome in cardiac surgery is more common than currently recognized [40,113]. This highlights the need for increased intraoperative vigilance, standardized reporting, and systematic post-event allergy evaluation to improve incidence estimates and enhance patient safety.

### 4.3. Etiology of Perioperative Kounis Syndrome

Chlorhexidine was the most frequently implicated agent in the reviewed literature, accounting for six cases of possible perioperative Kounis syndrome [45,46,47,52,114]. It is increasingly recognized as a significant trigger of severe perioperative hypersensitivity reactions in cardiac surgery, with multiple reports linking its use in skin antisepsis, urinary catheterization, vascular access—particularly central venous catheters—and other intravascular devices to anaphylaxis complicated by Kounis syndrome [45,46,47,52,65,114,115]. A distinguishing feature of chlorhexidine-induced reactions is the potential for persistent antigen exposure during the postoperative period, especially in the intensive care unit, if the source is not promptly identified and removed [45,46,52,61,114,116,117]. This continuous exposure may sustain mast cell activation and contribute to recurrent or refractory hypersensitivity reactions. In addition, limited awareness of chlorhexidine-containing materials among healthcare providers may delay recognition of the underlying cause [45,46,52,61]. From a clinical perspective, these observations underscore the importance of early identification and immediate removal of the offending source, as ongoing exposure may perpetuate mediator release and lead to repeated or prolonged episodes of instability [61]. This observation highlights the clinical importance of persistent antigen exposure, particularly from indwelling or coated medical devices, which may lead to recurrent or refractory hypersensitivity reactions if not promptly recognized and removed.

Two perioperative cases of Kounis syndrome associated with protamine were identified, including one definite and one possible case [49,118]. Protamine sulfate is a well-recognized trigger of hypersensitivity reactions in cardiac surgery due to its routine use for heparin reversal following cardiopulmonary bypass. Clinically, protamine may induce a wide range of immunologic adverse reactions, including thrombocytopenia, rash, bronchoconstriction, non-cardiogenic pulmonary edema, pulmonary vasoconstriction with right ventricular failure, systemic vasodilation with hypotension (vasoplegia), myocardial depression, arrhythmias, and cardiac arrest [58,119,120,121]. In the presence of cardiac manifestations—particularly sudden hemodynamic collapse or new echocardiographic abnormalities—the possibility of perioperative Kounis syndrome should be actively considered [49,118]. Although further research is needed to better characterize protamine-induced hypersensitivity, several risk factors have been proposed, including prior exposure to protamine, use of neutral protamine Hagedorn (NPH) insulin, fish allergy, vasectomy, and rapid administration of large doses [58,66,109,118,121]. These factors may contribute to immune sensitization and increase susceptibility to severe perioperative reactions.

Antibiotic-induced Kounis syndrome was identified in only one reported case following the administration of cefuroxime, likely reflecting the limited number of available reports rather than a true low incidence [42]. Nevertheless, antibiotics are widely used in cardiac surgery, and substantial evidence supports their association with perioperative anaphylaxis and anaphylactoid reactions.

Commonly used agents for antibiotic prophylaxis in cardiac surgery include cefazolin, cefuroxime, vancomycin, clindamycin, quinolones, and teicoplanin, with selection guided by local antimicrobial susceptibility patterns and individual allergy history [122,123,124]. Among these, cefazolin has been consistently reported as the most frequent cause of perioperative anaphylaxis in several populations, including those in the United States, France, Japan, and Spain [39,98,100,101]. Other agents, such as cefuroxime, quinolones, vancomycin, and teicoplanin, are also well-recognized triggers of perioperative hypersensitivity reactions [61,101,125]. The involvement of these antibiotics in the pathogenesis of Kounis syndrome is well documented [102,126,127,128,129,130]. However, the limited number of reported surgical cases—particularly in cardiac surgery—restricts the available evidence and may contribute to the apparent underrepresentation of certain agents, such as cefazolin, in Kounis syndrome compared with perioperative anaphylaxis [43,48]. From a clinical perspective, this discrepancy likely reflects underrecognition rather than a true difference in incidence. Given the routine administration of antibiotic prophylaxis, even rare hypersensitivity reactions may have significant clinical implications. These observations underscore the importance of accurate allergy assessment, appropriate antibiotic selection, and heightened perioperative vigilance. Further research is required to clarify the relationship between antibiotic exposure—particularly cefazolin and vancomycin, which are commonly used for prophylaxis against methicillin-sensitive and methicillin-resistant *Staphylococcus aureus*, respectively—and the pathogenesis of Kounis syndrome in cardiac surgery.

Although antibiotic-induced Kounis syndrome was identified in a limited number of reported cases, this likely reflects underrecognition rather than a true low incidence. Given the widespread use of antimicrobial prophylaxis in cardiac surgery, even rare hypersensitivity reactions may have significant clinical implications. This highlights the importance of accurate allergy assessment, appropriate antibiotic selection, and heightened intraoperative vigilance when administering antimicrobial agents.

Only one published case of possible perioperative Kounis syndrome associated with rocuronium has been reported, although another case of definite Kounis syndrome may have been related to either rocuronium or isoflurane [43,48]. This observation contrasts with epidemiological data identifying neuromuscular blocking agents (NMBAs) as frequent triggers of perioperative anaphylaxis; indeed, several studies suggest that they represent the most common cause, although this varies across institutions and geographic regions [61,86,92,131,132]. Non-depolarizing NMBAs, particularly rocuronium, are more commonly associated with hypersensitivity reactions compared with depolarizing agents such as succinylcholine or reversal agents such as sugammadex [33,133,134]. Taken together, these findings suggest that NMBA-related Kounis syndrome may be underrecognized or underreported in cardiac surgery. Clinically, NMBA-induced anaphylactic reactions typically occur during the induction phase of anesthesia, allowing early detection of cardiovascular and respiratory abnormalities through intraoperative monitoring [33,135]. In contrast, sugammadex-induced reactions tend to occur later, often shortly after extubation, reflecting its administration at the end of the procedure [135,136]. Additionally, cross-reactivity among NMBAs is well documented and may further complicate perioperative management [133,137].

No cases of latex-induced perioperative Kounis syndrome were identified in the reviewed literature, despite the well-established association between latex exposure and perioperative anaphylaxis [103,138,139,140,141]. Latex-related anaphylaxis typically occurs intraoperatively following exposure to medical equipment such as surgical gloves, masks, endotracheal tubes, ventilatory devices, and catheters [103,104,139,142]. Historically, latex hypersensitivity was more prevalent due to its widespread use for the prevention of bloodborne pathogen transmission. However, increased awareness and the implementation of preventive strategies have led to a significant decline in its incidence in recent decades [38,61,103,105,141]. Several risk factors for latex hypersensitivity have been identified, including a history of atopy, acral dermatitis, food allergy, neural tube defects, multiple prior surgical procedures, chronic healthcare exposure, and occupational exposure among healthcare workers [58,124,139]. The absence of reported latex-related Kounis syndrome cases in this review likely reflects reduced exposure and improved preventive measures rather than a lack of pathogenic potential.

Re-exposure to the causative agent appears to be a key mechanism underlying recurrence, emphasizing that early identification of the trigger is not only diagnostic but also a critical therapeutic and preventive priority. From a clinical perspective, antimicrobial agents represent modifiable perioperative risk factors, reinforcing their importance in both prevention and risk stratification.

### 4.4. Clinical Presentation and Diagnostic Investigation

Patients in this study exhibited heterogeneous, multisystem involvement, which was often subtle, as most hypotensive episodes occurred intraoperatively. Cardiovascular manifestations were universal and included hemodynamic instability, hyperdynamic circulation, regional wall motion abnormalities, biventricular dysfunction, Takotsubo-like cardiomyopathy, angiographic evidence of coronary vasospasm, elevated troponin levels, arrhythmias (bradycardia, tachycardia, atrial fibrillation), ST-segment elevation or depression, and cardiac arrest.

Respiratory involvement was observed in a subset of patients, either through clinical manifestations—such as bronchospasm, dyspnea, and hypoxemia—or via intraoperative monitoring findings, including decreased end-tidal carbon dioxide (EtCO_2_) and increased airway pressures. Mucocutaneous manifestations, including rash and angioedema, were reported in some cases and, when present, increased clinical suspicion of an underlying hypersensitivity reaction.

Perioperative cases of Kounis syndrome in cardiac surgery—highlighting suspected triggers, timing of exposure, clinical presentation, management strategies, and outcomes—are summarized in Table 2.

The clinical presentation of Kounis syndrome in cardiac surgery is often atypical and strongly influenced by operative conditions, particularly the use of general anesthesia and cardiopulmonary bypass (CPB). Classical respiratory and mucocutaneous signs of hypersensitivity reactions are frequently absent or subclinical, making intraoperative diagnosis more challenging [37,61,143]. Instead, perioperative Kounis syndrome commonly presents with sudden cardiovascular deterioration, including abrupt vasoplegia, a rapid increase in pulmonary arterial pressure, arrhythmias, or unexpected ventricular dysfunction occurring shortly after exposure to a potential allergen [61,144,145,146].

In off-pump and minimally invasive cardiac procedures, the clinical presentation is typically more apparent. Abrupt hypotension, bronchospasm, tachyarrhythmias, and ischemic electrocardiographic changes may develop within minutes of allergen exposure, reflecting preserved autonomic and vascular responses in the absence of extracorporeal circulation. In these settings, the temporal relationship between exposure and cardiovascular instability is often clearer, facilitating earlier recognition of Kounis syndrome.

The diagnostic approach to suspected Kounis syndrome in cardiac surgery must address both components of the syndrome simultaneously: acute hypersensitivity and myocardial ischemia [147]. Given the rapid and potentially life-threatening evolution of perioperative presentations, diagnosis is primarily clinical and relies on the integration of clinical findings with hemodynamic, imaging, and laboratory data, rather than on any single diagnostic modality [29,30,113,147,148].

Initial evaluation should include a 12-lead electrocardiogram (ECG), arterial blood gas analysis, and focused transesophageal echocardiography (TEE). Continuous ECG monitoring enables early detection of ischemic changes and malignant arrhythmias, while arterial blood gas analysis may reveal hypoxemia or metabolic disturbances associated with circulatory shock. TEE plays a central role in intraoperative recognition, as echocardiographic abnormalities often precede electrocardiographic changes [149,150,151].

The sudden appearance of new regional wall motion abnormalities, global ventricular dysfunction, hyperdynamic ventricles, apical ballooning, or acute right ventricular dilation—particularly following exposure to known triggers such as protamine or perioperative antibiotics—may represent early manifestations of allergic myocardial injury and should not be dismissed as nonspecific CPB-related dysfunction [41,44,152,153]. Beyond diagnosis, TEE provides essential real-time guidance for intraoperative management. It facilitates differentiation between left- and right-sided ventricular failure, identification of acute pulmonary hypertension (e.g., in protamine-related reactions), and dynamic assessment of myocardial response to therapy. These insights are critical for timely escalation of care, including consideration of CPB reinstitution, coronary angiography, or mechanical circulatory support.

Laboratory confirmation of mast cell activation relies primarily on serum tryptase measurement, which remains the biochemical cornerstone for diagnosing perioperative anaphylaxis. Recommended sampling includes an acute-phase specimen obtained 30–120 min after symptom onset and a baseline sample collected at least 24 h later. Results are interpreted using the validated criterion “1.2 × baseline + 2 μg/L” [138,148]. This approach improves diagnostic specificity and helps distinguish true anaphylaxis from nonspecific perioperative inflammatory or stress-related elevations commonly observed during CPB.

Cardiac biomarkers, such as serial high-sensitivity troponin measurements, may assist in quantifying myocardial injury in the postoperative period but have limited real-time utility intraoperatively [75]. Additional findings, including eosinophilia and elevated total immunoglobulin E (IgE), may further support the diagnosis of an underlying allergic process [28,154].

When ischemic changes persist on electrocardiography (ECG), ventricular dysfunction fails to resolve, or hemodynamic instability continues despite initial supportive therapy, emergent coronary angiography should be considered [147]. Less invasive alternatives include computed tomography angiography, single-photon emission computed tomography, and contrast-enhanced magnetic resonance imaging [75,113,153,154,155,156,157]. Coronary angiography remains essential to exclude fixed coronary obstruction and to identify dynamic vasospasm or thrombotic complications [147,154].

Identification of the causative allergen was achieved in eight patients in this study, most commonly through skin testing for multiple perioperative agents [41,45,46,48,50,52]. In two additional cases, the diagnosis was established through specific serological testing [51,52]. Patients with suspected perioperative hypersensitivity reactions—including anaphylaxis and Kounis syndrome—should undergo a structured post-event evaluation after clinical stabilization to identify the responsible trigger. Accurate identification of the allergen is critical for implementing preventive strategies in future procedures and reducing the risk of recurrence [141,158]. This is particularly important in patients requiring repeat surgery after an initial procedure was aborted due to a life-threatening allergic reaction [45,46,47,48].

Diagnostic testing should be tailored to the patient’s exposure history and include all potential perioperative allergens, such as prophylactic antibiotics, chlorhexidine, neuromuscular blocking agents, latex, and protamine [33,38,108,141]. Skin testing—initially with prick testing followed by intradermal testing—is typically the first-line diagnostic approach and should be performed 4–6 weeks after the reaction to ensure optimal sensitivity and specificity [33,100,108,141,159]. Measurement of specific immunoglobulin E (IgE) is particularly useful in the evaluation of latex and chlorhexidine allergy, although its role in other drug-related reactions remains less well established. Additional diagnostic modalities, including the basophil activation test and drug provocation testing, may provide complementary information in selected cases [33,100,108,141,159]. Drug provocation testing may be considered when the etiology remains unclear and the anticipated risk is acceptable, with careful consideration of the dose and clinical context.

**Figure 2:**
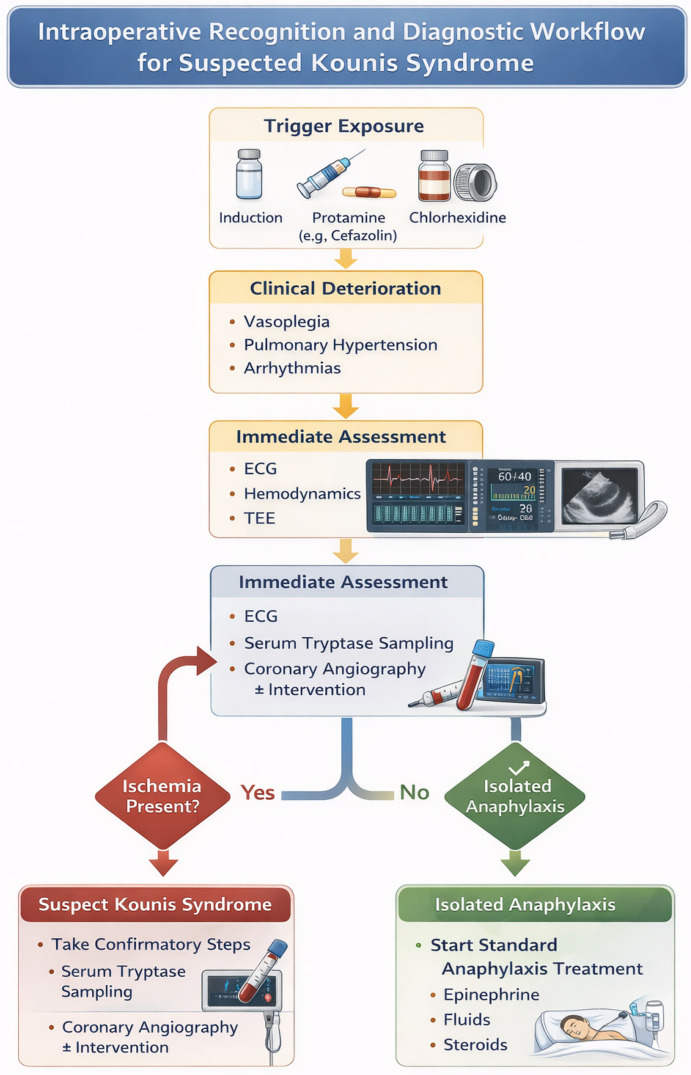
Intraoperative recognition and diagnostic workflow for suspected Kounis syndrome.

Distinguishing isolated anaphylactic shock from Kounis syndrome in the perioperative setting is essential (Table 3). Although both conditions may present with abrupt hypotension and cardiovascular collapse, they differ significantly in pathophysiology and management [41]. Early recognition of myocardial ischemia—supported by ECG findings and intraoperative transesophageal echocardiography (TEE)—helps differentiate coronary involvement from isolated anaphylaxis and guides appropriate escalation of care. The differential diagnosis also includes type 1 acute myocardial infarction, Takotsubo cardiomyopathy, hypersensitivity myocarditis, hypereosinophilic syndrome, mastocytosis, and vasoplegia [40,49,118,155,159,160]. To facilitate clinical application, a structured intraoperative diagnostic workflow is proposed, integrating hemodynamic monitoring, transesophageal echocardiography, electrocardiographic findings, and biochemical markers to support early recognition of Kounis syndrome. Such algorithm-based approaches may improve diagnostic accuracy and reduce delays in the management of this underrecognized condition. A practical intraoperative recognition and diagnostic workflow for suspected Kounis syndrome is summarized in Figure 2.

### 4.5. Management: Dual-Pathway Approach

The management of Kounis syndrome in cardiac surgery requires a dual-pathway approach that simultaneously addresses life-threatening anaphylaxis and acute coronary ischemia. Immediate discontinuation of the suspected triggering agent is essential [161,162]. Epinephrine remains the cornerstone of treatment for severe anaphylaxis and should be administered promptly, with careful titration to restore systemic perfusion while minimizing the risk of exacerbating coronary vasospasm, arrhythmias, and Takotsubo-like cardiomyopathy [152,163,164]. Adjunctive measures include supplemental oxygen, fluid resuscitation, administration of H1 and H2 antihistamines, bronchodilators, and corticosteroids to attenuate the inflammatory response [152,163]. In patients receiving β-blockers, glucagon may be required to treat refractory hypotension and bradycardia [163,165,166].

Targeted management of the ischemic component is equally critical. Coronary vasospasm may be treated with intravenous nitrates and/or calcium channel blockers, provided that hemodynamic stability allows their safe use [163]. Morphine should be avoided due to its potential to induce histamine release and exacerbate allergic reactions; in contrast, synthetic opioids such as fentanyl are less likely to trigger mast cell activation [163,167]. β-blockers should be used with caution during active coronary spasm, as unopposed α-adrenergic stimulation may worsen vasoconstriction. These considerations highlight the need for individualized therapy guided by continuous hemodynamic and echocardiographic monitoring [168,169,170]. Venoarterial extracorporeal membrane oxygenation (VA-ECMO) and ventricular assist devices (VAD) have been reported as effective rescue strategies in refractory shock, providing time for resolution of the inflammatory response and myocardial recovery [43,118,171,172,173].

Specific management strategies may be required depending on the underlying trigger. Ongoing exposure to the causative agent should be promptly terminated [52,161,162]. For example, chlorhexidine-coated central venous catheters should be removed when implicated. Sugammadex may bind neuromuscular blocking agents and potentially mitigate their effects; however, it may also trigger hypersensitivity reactions due to its molecular structure [174,175]. In cases of severe protamine-induced reactions unresponsive to conventional therapy, emergent re-heparinization and reinstitution of cardiopulmonary bypass may be necessary to restore hemodynamic stability and myocardial perfusion [49].

When myocardial ischemia persists or electrocardiographic findings consistent with ST-elevation myocardial infarction (STEMI) develop, urgent percutaneous coronary intervention (PCI) is indicated. Intracoronary administration of nitrates during angiography may provide both diagnostic and therapeutic benefits by confirming dynamic coronary vasospasm and achieving rapid resolution [28,163]. This approach is particularly useful in differentiating myocardial infarction with non-obstructive coronary arteries (MINOCA) from fixed coronary obstruction and in guiding subsequent management decisions.

**Figure 3:**
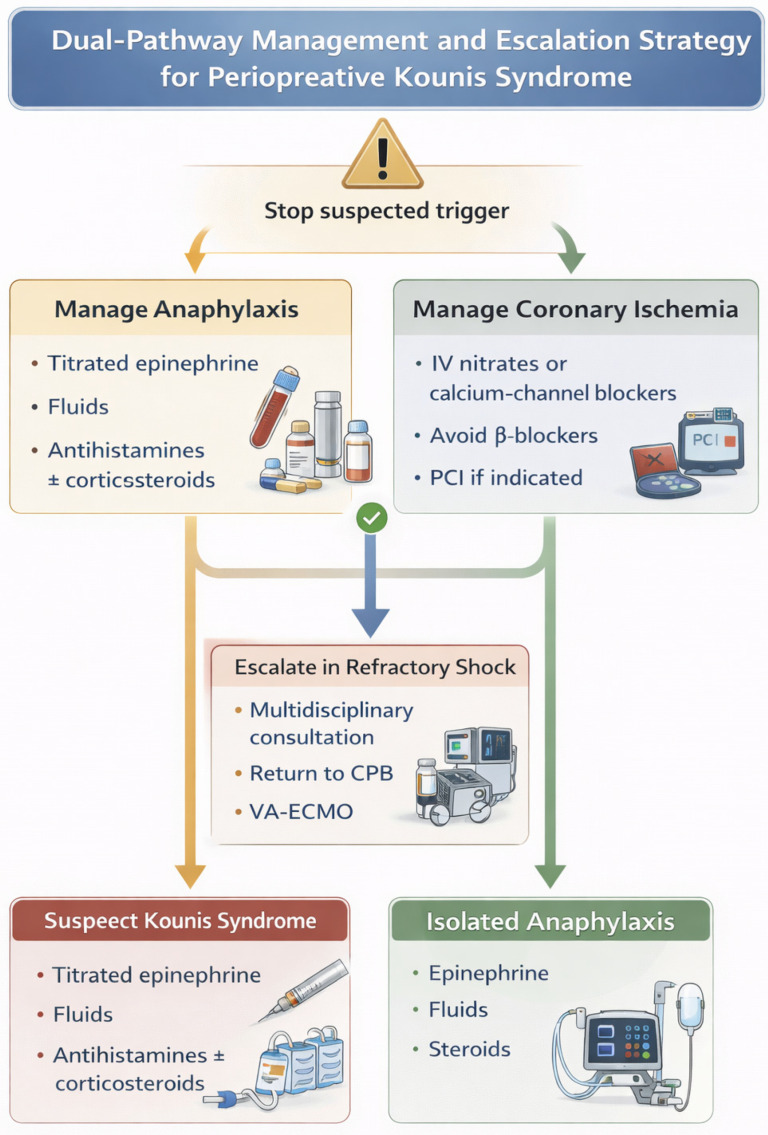
Dual-pathway management and escalation strategy for perioperative Kounis syndrome.

A pragmatic, surgery-oriented management framework for perioperative Kounis syndrome is outlined in Table 4. From a practical perspective, combining a structured diagnostic algorithm with a dual-pathway management strategy may facilitate timely intervention and improve perioperative outcomes in this challenging clinical scenario. The dual-pathway management approach and escalation options for perioperative Kounis syndrome are illustrated in Figure 3.

### 4.6. Outcomes and Prevention

Overall, patient outcomes in this study were predominantly favorable. This observation likely reflects prompt recognition and timely management of perioperative complications in the operating room or early postoperative period. Only one fatal case was reported, involving a critically ill patient with a surgical site infection, in whom the clinical course was likely exacerbated by possible Kounis syndrome despite aggressive management [51].

Clinical outcomes in perioperative Kounis syndrome are strongly influenced by the timeliness of diagnosis and the implementation of structured, protocol-driven management strategies [33,34,123]. Early recognition of concurrent allergic and ischemic manifestations, combined with coordinated multidisciplinary management involving anesthesiology, surgery, and cardiology, is associated with improved hemodynamic stabilization and myocardial recovery. Cardiac surgery programs that incorporate predefined escalation pathways—including early consideration of cardiopulmonary bypass reinstitution and rapid access to mechanical circulatory support—have reported favorable outcomes even in severe presentations complicated by refractory shock or significant ventricular dysfunction [43,176].

Prevention represents a key component of risk reduction and relies primarily on thorough preoperative hypersensitivity assessment. This includes targeted evaluation for prior reactions or sensitization to protamine (particularly in patients with prior exposure to neutral protamine Hagedorn insulin), β-lactam antibiotics, chlorhexidine, neuromuscular blocking agents, latex, and iodinated contrast media. In patients with known chlorhexidine hypersensitivity, strict avoidance of chlorhexidine-containing products—including skin preparations and impregnated medical devices—is essential [105].

Additionally, careful perioperative strategies may further reduce risk. For example, slow or graded administration of protamine under close hemodynamic and echocardiographic monitoring has been associated with a lower incidence and severity of adverse reactions during heparin reversal. These preventive measures, combined with heightened perioperative vigilance, are critical for minimizing complications and improving patient safety.

### 4.7. Knowledge Gaps and Future Directions

Despite increasing recognition, significant knowledge gaps remain. The true incidence of Kounis syndrome in cardiac surgery is likely underestimated, as cardiopulmonary bypass (CPB) may obscure dermatologic and hemodynamic manifestations, and routine measurement of biomarkers such as serum tryptase is not consistently performed. Prospective registries incorporating standardized diagnostic criteria, systematic biomarker assessment, and intraoperative imaging endpoints are needed to more accurately define epidemiology and clinical outcomes [177].

Future research should focus on optimizing therapeutic strategies during CPB, particularly the balance between epinephrine administration and coronary vasodilators, as well as on the development and validation of risk stratification tools for high-risk agents such as protamine and chlorhexidine. Emerging diagnostic approaches—including skin testing (prick and intradermal), basophil activation testing, and component-resolved diagnostics—may improve preoperative risk assessment and patient stratification [36,95].

In addition, evidence from contrast-associated Kounis syndrome and interventional cardiology highlights the importance of institutional preparedness, including rapid access to coronary angiography and mechanical circulatory support, as key determinants of patient survival [30,66,176,178].

## 5. Limitations

This review has several limitations that should be considered when interpreting its findings. First, the available evidence on Kounis syndrome in cardiac surgery is largely derived from case reports, case series, perioperative audits, and narrative reviews, with a notable absence of large observational or randomized studies. Consequently, conclusions regarding incidence, risk stratification, and optimal management strategies are primarily based on observational data and expert consensus rather than high-level evidence.

Second, the true incidence of perioperative Kounis syndrome is likely underestimated, particularly in the context of cardiopulmonary bypass (CPB). CPB may obscure classical dermatologic and respiratory manifestations of hypersensitivity, while hemodynamic instability and myocardial dysfunction are often attributed to alternative perioperative causes. In addition, inconsistent use of confirmatory biomarkers—particularly serum tryptase and cardiac troponins—along with the lack of standardized intraoperative diagnostic protocols, further limits accurate case identification and reporting [179,180].

Third, heterogeneity across published reports represents an additional limitation. Variability in patient populations, surgical procedures, anesthetic techniques, trigger exposure, diagnostic criteria, and outcome definitions complicates direct comparisons and precludes meaningful quantitative synthesis. This heterogeneity also limits the identification of clear prognostic indicators and the development of standardized therapeutic algorithms applicable across different cardiac surgery settings [176].

Fourth, although this review focuses on cardiac surgery, several concepts are extrapolated from related fields such as immunology and cardiology, where intraoperative decision-making is less directly addressed. As a result, certain aspects of surgical management—such as decisions regarding cardiopulmonary bypass reinstitution, mechanical circulatory support (e.g., ventricular assist devices), or invasive coronary evaluation—remain insufficiently defined in the current literature.

Finally, as a narrative review, this work is inherently subject to selection bias and does not follow a systematic review or meta-analytic methodology. Although efforts were made to comprehensively search PubMed-indexed literature and prioritize clinically relevant studies, unpublished data and non-indexed sources were not included. Future prospective registries and standardized reporting frameworks are needed to refine risk stratification, validate diagnostic approaches, and optimize management strategies for Kounis syndrome in cardiac surgery.

## 6. Key Messages for Cardiac Surgeons

1. Kounis syndrome should be suspected when perioperative anaphylaxis and ischemia coexist, particularly after the administration of common allergens, such as antibiotics, NMBAs, chlorhexidine, and protamine.
2. CPB masks allergic signs; thus, electrocardiographic and echocardiographic monitoring is often the earliest diagnostic tool.
3. Management requires a dual-pathway approach to anaphylaxis and ischemia.
4. Early return to CPB or VA-ECMO is essential for refractory cases.
5. Post-event allergy investigation is essential to prevent recurrence, especially

## 7. Conclusions

Kounis syndrome should be considered whenever perioperative anaphylaxis coincides with myocardial ischemia, particularly following chlorhexidine antisepsis, protamine for anticoagulation reversal in CPB, antibiotic prophylaxis, and induction of general anesthesia. In cardiac surgery, CPB may mask classical allergic manifestations, necessitating a high index of suspicion. Early recognition, balanced pharmacologic management, and timely imaging or reperfusion strategies are necessary to improve outcomes. These conclusions should be interpreted with caution, given the small number of reported cases and the heterogeneity of the available evidence, which is largely derived from case reports and case series. Although this review does not allow estimation of the true incidence of Kounis syndrome in cardiac surgery, the available evidence suggests that the condition is likely underrecognized and underreported. This demonstrates the need for structured perioperative diagnostic and management protocols to improve detection and patient outcomes. The implementation of structured diagnostic and management algorithms may further enhance perioperative recognition and improve patient outcomes. Multidisciplinary collaboration and tailored post-event allergy evaluation could prevent recurrence during reoperation or future procedures.

## Figures and Tables

**Table 1:** Summary of reported cases of perioperative Kounis syndrome in cardiac surgery.

Category	Definite Kounis Syndrome (*n* = 5)	Possible Kounis Syndrome (*n* = 10)
Diagnostic criteria	Anaphylaxis + documented myocardial ischemia	Anaphylaxis + severe cardiovascular involvement
Cardiac arrest	2/5	7/10
Cardiovascular collapse	-	3/10
Common triggers	Chlorhexidine, protamine, antibiotics	Chlorhexidine, protamine, anesthetic agents, colloids
ECG changes	Frequent (ST elevation/depression)	Hyperdynamic ventricles, dysfunction
Echocardiographic findings	RWMA, ventricular dysfunction	Hyperdynamic ventricles, dysfunction
Biomarker elevation	3/5	Limited data
Recurrence	Present in selected cases	Frequent with re-exposure
Treatment	Adrenaline, vasopressors, CPB/ECMO	Similar approach
Outcome	Mostly survival	Mostly survival, 1 fatal case
Clinical note	Clear ischemic component	Diagnostic uncertainty

Abbreviations: ECG, electrocardiogram; RWMA, regional wall-motion abnormality; CPB, cardiopulmonary bypass; ECMO, extracorporeal membrane oxygenation.

**Table 2:** Reported cases of perioperative Kounis syndrome in cardiac surgery.

Author, Year	Age (Years)/Sex (M/F)	Type of Surgery	Trigger	Onset	Clinical Presentation and Diagnosis	Treatment	Outcome and Follow-Up
A. Cases described as perioperative KS in cardiac surgery							
Kumaran, et al., 2023 [42]	56/M	Elective CABG	Cefuroxime	Intraoperative	Tachycardia, hypotension, lack of rash, decreased EtCO_2_, increased PIP, increased tryptase/eosinophils, ST elevation (ECG), cardiac arrest (V-fib)	100% oxygen, crystalloids, Trendelenburg position, diphenhydramine, hydrocortisone, inhaled β_2_ agonist, CPR, CPB, adrenaline, dobutamine, noradrenaline	Survival Successful completion of surgery with improved cardiac function n/a
García, et al., 2018 [41]	69/M	Elective MVR	Amiodarone	Intraoperative	Hypoxemia, hypotension, lack of rash, decreased EtCO_2_, increased PIP, increased tryptase/CK-MB/troponin, ST elevation (EGC), biventricular dysfunction (TEE) Positive prick test for amiodarone	Crystalloids, phenylephrine, adrenaline, salbutamol, CPB, noradrenaline, hydrocortisone, methylprednisolone, FFP, pRBCs, dobutamine, furosemide	Survival Successful completion of surgery Normal echocardiogram (after 7 days)
Parent, et al., 2011 [43]	2/M	Elective repair of ASD	n/a	Intraoperative and postoperative (POD 0)	Intermittent periods of diffuse ST elevation consistent with incipient ischemia (intraoperative) Hypoxemia, bradycardia, hypotension, lack of rash, cardiac arrest, anuria, compartment syndrome, watershed stroke, seizures, ARDS, increased troponin/tryptase/IgE, ST elevation (ECG), biventricular dysfunction (echo), narrowed coronary vessels (angiography), normal cardiac biopsy consistent with Kounis syndrome (POD 0)	Intubation, mechanical ventilation, inotropes, CPR, ECMO, nitroglycerin, BiVAD, hemodialysis, fasciotomy, famotidine, cromolyn	Survival Successful completion of surgery with improved cardiac function. Urgent implantation of BiVAD, which was later removed. Normal echocardiogram (after 12 months)
Li, et al., 2014 [118]	37/F	Elective MVR	Protamine	Intraoperative	Hypotension, lack of rash, elevated CVP, decreased cardiac index, increased troponin, ST elevation (ECG), lack of complications in the surgical field, ballooning of the LV apex (TTE), normal angiography	Adrenaline, diphenhydramine, vasopressin, desmopressin, levothyroxine, hydrocortisone, methylene blue, noradrenaline, phenylephrine, ECMO	Survival Successful completion of surgery with improved cardiac function. Emergent sternal reopening for exclusion of cardiac tamponade. Normal ECG and TTE
Cheung, et al., 2016 [44]	79/F	Elective MVR	Amiodarone	Intraoperative	Hypotension, lack of rash, inferolateral wall hypokinesis (TEE), vasoconstriction of the distal coronary branches (angiography)	Phenylephrine, vasopressin, calcium chloride, adrenaline	Survival Successful completion of surgery
Β. Additional possible cases of perioperative Kounis syndrome in cardiac surgery (anaphylaxis and cardiac arrest)							
Zhou, et al., 2019 (commented by Kounis et al., 2019) [45,114]	59/M	Elective orthotopic heart (2nd operation) transplantation 13 months following elective implantation of LVAD (1st operation)	Chlorhexidine	Intraoperative and postoperative	Pruritic irritation due to skin application, probably in the context of sensitization (preoperative night) Hypotension, tachycardia, wheezing, urticaria, angioedema, normal tryptase in the context of anaphylaxis (1st operation and postoperative) Shock, erythema, and cardiac arrest, probably in the context of Kounis syndrome (2nd operation) Positive prick testing for chlorhexidine and ceftriaxone (6 weeks after LVAD implantation)	Adrenaline, diphenhydramine, and hydrocortisone (1st operation). CPR, adrenaline, noradrenaline, vasopressin (2nd operation).	Survival Successful completion of LVAD implantation Unsuccessful completion of heart transplantation after 13 months, but it was completed successfully 5 months later
Stephens, et al., 2001 [46]	50/M	Elective CABG 3 weeks after previous CABG	Chlorhexidine	Intraoperative	Possible history of dermatitis to chlorhexidine Hypotension, erythema, angioedema, increased tryptase/CRP, normal complement (1st operation) Rash, angioedema, hypotension, increased tryptase, ST depression/A-fib/V-fib (ECG), PEA, increased tryptase, normal CK/CK-MB (2nd operation) Positive skin testing for chlorhexidine 4% and 0.4% (after the first operation) Positive skin testing for chlorhexidine 4%, 0.4%, and 0.04% (after the second operation)	CPR, 100% O_2_, phenylephrine, adrenaline, crystalloids, calcium, chlorpheniramine, hydrocortisone, ranitidine (1st operation) Ineffective premedication with corticosteroids and antihistamines. CPR, phenylephrine, adrenaline, noradrenaline, crystalloids, calcium, lignocaine, aprotinin, chlorpheniramine, cardioversion (2nd operation)	Survival Unsuccessful completion of both CABG procedures, leading to medical management of angina
Jaroenpuntaruk, et al., 2025 [47]	75/M	Elective CABG repeated 10 weeks later	Chlorhexidine	Intraoperative	Hypotension progressing to PEA, lack of rash, increased tryptase, normal troponin, normal TEE, normal chest CTA (1st operation) Tachycardia, hypotension, flushing (2nd operation) Positive skin testing for chlorhexidine (6 weeks after the first operation)	CPR, adrenaline, vasopressors (1st operation) Adrenaline (2nd operation)	Survival Unsuccessful completion of the first CABG, but successful completion of the second CABG
Macharadze, et al., 2020 [48]	66/M	Elective CABG and AVR	Rocuronium	Intraoperative	Bronchospasm, hypoxemia, tachycardia, hypotension progressing to cardiac arrest, flushing Positive skin testing for rocuronium (prick), vecuronium (intradermal), and pancuronium (intradermal) (6 weeks after first operation)	CPR, intubation, crystalloids, pRBCs, albumin, metaraminol, adrenaline, sugammadex, ECMO, vasopressin, noradrenaline, emergent bronchoscopy for secretion clearance, ipratropium, salbutamol	Survival Unsuccessful completion of the first surgery, but successful completion of the second surgery
Ripoll, et al., 2019 [49]	69/M	Elective repair of the left anterior descending artery bridging	Protamine	Intraoperative and postoperative (POD 2 and 4)	Intraoperative episode of hypotension consistent with anaphylaxis Postoperative episode of hypotension, coagulopathy, lack of rash, increased chest tube drainage, prominent soft tissue bleeding consistent with hemorrhagic shock (POD 0) Postoperative episode of hypoxemia, hypotension, cardiac arrest, soft tissue bleeding, coagulopathy, lack of rash, hyperdynamic ventricles (TTE) consistent with mixed shock (hemorrhage-Kounis syndrome) (POD 2) Postoperative episode of respiratory distress, hypotension, flushing, elevated tryptase, hyperdynamic ventricles (TTE), systemic mastocytosis (bone marrow biopsy) consistent with anaphylaxis (POD 4)	CPB, adrenaline, noradrenaline, dexamethasone, diphenhydramine (intraoperative) Mediastinal exploration with hemorrhage control, vasopressors (POD 0) Intubation, mechanical ventilation, adrenaline, phenylephrine, calcium, crystalloids, CPR, emergent sternotomy, transfusions, vasopressors, protamine after pretreatment with antihistamines/corticosteroids (POD 2) CPAP, vasopressor, corticosteroids, antihistamines (POD 4)	Survival Successful completion of surgery Two emergent surgeries for the investigation of postoperative hemorrhage (POD 0 and 2)
Komericki, et al., 2014 [50]	40/M	Elective MVR	Human serum albumin	Intraoperative and postoperative	Intraoperative episode of hypotension progressing to cardiac arrest with a clear surgical field Postoperative episode of hypotension, dyspnoea, rash, normal tryptase, increased IgE (POD 2) Positive skin testing for Haemocomplettan P leading to positive skin testing (prick and intradermal) for “human albumin 20%, CSL Behring” and “human albumin 20%, Octapharma, Vienna, Austria”	CPR, crystalloids, colloids, emergent resternotomy with direct cardiac massage, adrenaline, prednisolone, dimetindene (intraoperative) Crystalloids, dimetindene, prednisolone (POD 2)	Survival Successful completion of surgery Emergent resternotomy for surgical field exploration and direct cardiac massage (POD 0)
Molina-Molina, et al., 2019 [51]	65/Μ	Repair of type A aortic dissection followed by surgical debridement	Gelatin colloid (Gelaspan)	Postoperative (POD 57)	Oropharyngeal pruritus, lack of rash, altered mental status, cardiac arrest (POD 57) Positive testing for tryptase and IgE specific to Gelaspan (post-mortem allergological work-up)	CPR, intubation, adrenaline, noradrenaline, isoprenaline, methylene blue	Death Successful completion of surgery complicated by surgical wound infection necessitating urgent surgical debridement (POD 57)
Baird, et al., 2019 (Case 1) [52]	71/M	Urgent MVR	Chlorhexidine	Intraoperative and postoperative (POD 0)	Intraoperative episode of hypotension progressing to cardiovascular collapse, increased airway pressure, abnormal end-tidal CO_2_, urticaria (intraoperative) Recurrent postoperative episodes of hypotension with increased tryptase (POD 0) Positive intradermal testing to 1:100 chlorhexidine	Adrenaline (intraoperative) Crystalloids (POD 0)	Survival Successful completion of surgery
Baird, et al., 2019 (Case 2) [52]	76/M	Urgent CABG	Chlorhexidine	Intraoperative	Intraoperative episode of hypotension progressing to cardiovascular collapse with rash and increased tryptase Positive prick testing for chlorhexidine	Adrenaline, phenylephrine, ephedrine, crystalloids	Survival Successful completion of surgery
Baird, et al., 2019 (Case 3) [52]	71/M	Urgent CABG	Chlorhexidine	Intraoperative and postoperative (POD 0)	Intraoperative episode of tachycardia, hypotension progressing to cardiovascular collapse with increased tryptase Postoperative episode of hypotension and angioedema Positive testing for specific IgE against chlorhexidine	Adrenaline, vasopressors, crystalloids Adrenaline, corticosteroids, antihistamines, vasopressors, inotropes	Survival Successful completion of surgery

**Table 3:** Diagnostic features and differential diagnosis of perioperative Kounis syndrome in cardiac surgery.

Feature	Kounis Syndrome	Isolated Perioperative Anaphylaxis	CPB-Related Myocardial Stunning/Low Output	Acute Coronary Occlusion (Non-Allergic)
Temporal relationship to exposure	Immediate or early (minutes) after allergen exposure (e.g., protamine, antibiotics, chlorhexidine)	Immediate after allergen exposure	Variable; often after CPB separation or reperfusion	Variable; not necessarily linked to allergen
Cutaneous manifestations	Often absent during CPB; may be subtle or delayed	Common (urticaria, flushing, angioedema)	Absent	Absent
Bronchospasm	May occur, but not universal	Frequent	Rare	Rare
Hemodynamic profile	Vasoplegia ± acute pulmonary hypertension; possible RV failure	Vasoplegia with distributive shock	Low cardiac output without vasoplegia	Cardiogenic shock
TEE findings	New regional wall-motion abnormalities; possible RV dilation/dysfunction	Usually normal or hyperdynamic ventricles	Global ventricular dysfunction	Territory-specific RWMA
ECG changes	Dynamic ST-segment changes, transient ischemia	Usually normal or nonspecific	Nonspecific ST-T changes	Persistent ST elevation or new Q waves
Serum tryptase	Elevated (acute rise above baseline)	Elevated	Normal	Normal
Coronary angiography	Coronary spasm ± allergic thrombus (Type III KS)	Normal coronaries	Normal coronaries	Fixed coronary obstruction
Response to nitrates	Often rapid improvement	No effect	No effect	Limited or none
Key diagnostic clue	Temporal link between allergy and ischemia	Systemic allergic signs without ischemia	CPB-related myocardial depression	Persistent ischemia unrelated to allergy

Abbreviations: CPB, cardiopulmonary bypass; ECG, electrocardiogram; KS, Kounis syndrome; RV, right ventricle; RWMA, regional wall-motion abnormality; TEE, transesophageal echocardiography.

**Table 4:** Practical management principles for perioperative Kounis syndrome in cardiac surgery.

Clinical Scenario	Immediate Actions	Targeted Therapy	Escalation Strategy
Suspected KS during induction or early intra-operative phase	Stop suspected trigger; secure airway; high-flow oxygen; invasive hemodynamic monitoring	Titrated epinephrine; IV crystalloids; H1/H2 antihistamines; corticosteroids	TEE assessment; serum tryptase sampling
Predominant coronary vasospasm	Hemodynamic stabilization; avoid β-blockers	IV nitrates ± calcium-channel blockers (if BP permits)	Urgent coronary angiography
Protamine-associated cardiovascular collapse	Immediately stop protamine; re-heparinize	RV support; pulmonary vasodilators; vasopressors as needed	Return to CPB
Persistent ischemia or STEMI pattern	Continuous ECG and TEE monitoring	Intracoronary nitrates; antiplatelet therapy as indicated	Urgent PCI
Refractory shock despite conventional therapy	Multidisciplinary escalation (surgery–anesthesia–cardiology)	Dual-pathway therapy (anaphylaxis + ischemia)	VA-ECMO
Post-event stabilization and prevention	Detailed documentation; avoid re-exposure	Allergy referral; targeted avoidance strategy	Protocol update for future procedures

Abbreviations: BP, blood pressure; CPB, cardiopulmonary bypass; ECG, electrocardiogram; KS, Kounis syndrome; PCI, percutaneous coronary intervention; RV, right ventricle; STEMI, ST-elevation myocardial infarction; TEE, transesophageal echocardiography; VA-ECMO, venoarterial extracorporeal membrane oxygenation.

## Data Availability

No new data were created or analyzed in this study. Data sharing is not applicable to this article.
